# The influence of firm’s feedbacks on user-generated content’s linguistic style matching–An explanation based on communication accommodation theory

**DOI:** 10.3389/fpsyg.2022.949968

**Published:** 2022-07-22

**Authors:** Dewen Liu, Chenyiming Gong, Sikang Zhang, Yongbin Ma

**Affiliations:** ^1^School of Management, Nanjing University of Posts and Telecommunications, Nanjing, China; ^2^School of Marxism, Shanghai University of Finance and Economics, Shanghai, China; ^3^College of Business, Shanghai University of Finance and Economics, Shanghai, China; ^4^Business School, Ningbo University, Ningbo, China

**Keywords:** firm’s feedbacks, posting behaviors, linguistic style matching, user-generated content, communication accommodation theory

## Abstract

In virtual brand communities, users and firms continuously use different or similar linguistic styles to communicate with each other. Existing literature has demonstrated that the linguistic style matching (LSM) between the coming users’ posts [user-generated content (UGC)] and existing firms’ content will influence users’ behavior, like promoting users to release more posts. However, little research has been conducted to analyze how firms’ feedbacking behaviors influence LSM. To fill the gap, this paper uses Python to measure the LSM between 69,463 posts from 9,777 users and existing firms’ generated content in the MIUI community and examines the impact of firms’ feedbacks on this LSM. The results show that the firms’ feedbacks frequency increased the LSM, but the firms’ feedbacks text length decreased the LSM. In addition, users’ textual sentiment and the published text length moderate the impact of firms’ feedbacks (e.g., frequency, text length) on LSM. Specifically, the users’ textual sentiment valence increases the positive effect of firms’ feedbacks frequency and weakens the negative effect of firms’ feedbacks text length on LSM. The users’ produced content text length reduced the positive effect of firms’ feedbacks frequency and offset the negative effect of the firms’ feedbacks text length on LSM. Further, the effects above are significant for the relatively active users but not for the inactive ones. Based on communication accommodation theory, this paper investigates the impact of firms’ feedbacks frequency and text length on subsequent users’ posting behaviors, providing an essential reference for guiding firms’ virtual brand community management.

## Introduction

As firms continue to transform digitally, virtual brand communities (VBCs) for product promotion and customer relationship management have become an everyday part of their marketing practices (Garay-Tamajón and Morales-Pérez, 2020; Yao et al., 2021). Customers’ knowledge, information, and content in VBCs [also called user-generated content (UGC)] become a new source of channel information for firms to collect customer suggestions and make innovations (Chen et al., 2019) as the data generated by average users reveals their fundamental needs (Saura et al., 2021). Among the UGCs’ traits, the language styles convey the customers’ personalities, thoughts, or attitudes (Xiang et al., 2022), so that firms who understand or influence these styles can gain better market insights and improve the coherence between users and firms (Zhong and Schweidel, 2020). For example, Xiaomi, a leading mobile phone manufacturer in China, has created a unique “Xiaomi style” (Cao et al., 2020), attracting many devotees to constantly contribute their knowledge to the virtual community or even improve the sales performance by deciphering the information cues (Ludwig et al., 2013). The language style in a dialog demonstrates the individual distinction in self-expression (Gonzales et al., 2010). Based on the assumption that language shows an individual’s perceptions of the world, if two individuals show linguistic style matching (LSM) in a dialog, it would signify that they are harmonious or congruent in psychological activities (Pennebaker et al., 2003). Some researchers have highlighted that importance of LSM in the online environment results from the cost advantage in improving conversion rate (Ludwig et al., 2013).

The previous studies devoted attention to the outcomes (e.g., conversion rates, review helpfulness) of LSM (Ludwig et al., 2013; Wang et al., 2019). For example, Ludwig et al. (2014) showed that the higher the LSM between users’ posts and existing posts, the higher the probability that users will continue to participate in the community. The LSM between the firm’s feedbacks and users’ posts also predicts users’ subsequent posting speed (Piezunka and Dahlander, 2019). However, users can freely design their posts and replies in VBCs (Zheng et al., 2022). The contents were not generated in the first place (Goes et al., 2014), and linguistic style uniformity was not innate either, but developed through gradual interactions with other users or the focal brands in VBCs. Unlike other virtual communities (e.g., Twitter), VBCs gathered users with common brand hobbies or consume experiences (Sicilia and Palazón, 2008), and they predominantly seek emotional support and individual-brand connections (Veloutsou and Mafe, 2020). Accordingly, the linguistic styles in VBCs have been extensively researched as an information signal or a genre of discourse (Liu et al., 2019; Cenni and Goethals, 2021), reflecting the users’ inferences about the focal brand (Jahng and Hong, 2017).

The studies on identifying the influential factors of LSM have been inchoate in recent years. Participants interact linguistically in VBCs to facilitate smooth communication between participants and foster users’ identification and loyalty to the brand (Santos et al., 2022). Based on topic modeling and linguistic styles, Xiang et al. (2022) explored the reasons for producing various topics when posting in VBCs and proposed the concept of users’ variety-producing behaviors. Wang et al. (2022) explored the corpus of the product-centered language style of female consumers in China. From these initial efforts, it is clear that academia intends to detail the antecedents of LSM between participators in the online conversations that synchronize that specific way of expression. However, it is unclear how the linguistic styles of firms and users interact with each other. Thus, the current research remains two unresolved questions in providing practical guidance to firms on influencing and leading the linguistic styles: (1) What explains the LSM between users and firms? (2) What can the firm do to utilize LSM? This study empirically approaches these two questions with a theoretical explanation based on communication accommodation theory.

Communication accommodation theory provides a potential lens to explain the intertwined two-way effect between users’ posts and feedbacks in VBCs (Chen et al., 2019). Firms can better understand users’ emotional states or predict users’ behaviors by revealing the textual information (e.g., tweets) hidden in the online environment through effective text analysis technologies (Saura et al., 2022). Existing literature on communication accommodation theory has demonstrated that people would automatically mimic others’ linguistic or verbal styles during the communication process (Gnisci, 2005). Like face-to-face communication, individuals also experience online virtual interactions (Wang et al., 2022) while posting and replying. Especially in VBCs, users share strong connections to the brand, and the firm’s feedback exerts great influence on users’ following content (Piezunka and Dahlander, 2019). Therefore, based on communication accommodation theory and the nature of VBCs, the reasons for LSM may be that users “mimic” or adjust their way of posting in contacting frims’ feedbacks. Previous works on communication accommodation theory mainly discussed the cross-influences of linguistic styles during communication between individuals, and whether this theory can be used to explain the LSM from firms’ side needs empirical evidence.

Above all, the originality of the present study is that, covering a gap in previous research of LSM and VBCs’ management. Based on communication accommodation theory (e.g., Giles, 2016), this paper investigates the link between the frims’ feedbacks and the LSM between the coming UGC (e.g., posts) and existing firm-generated content (e.g., firms’ feedbacks) focused on textual posts of MIUI as the source of data, as well as to explore the moderators that “change” the relationships. This paper aims to understand how firms’ feedback behavior affects users’ linguistic style, which was done by analyzing daily textual data and calculating the matching scores.

Regarding methodology, the approach adopted in the present study unfolds in the following two steps. First, we reviewed the papers related to VBCs and linguistic styles. Second, we used the data-mining technique and textual analysis (TA) to compute LSM as a statistical measure that reflects the similarity in topics between firms’ feedbacks and users’ posts. Recently, TA has been applied in analyzing textual data to capture individuals’ cognition, emotion, and intention in the virtual world (Saura et al., 2021, 2022). Based on the procedures, we found that firms’ feedbacks frequency and length have opposite impacts on the LSM, and the features (e.g., sentiment valence, content length) of users’ posts moderate the relationships.

The marginal contributions of this paper are as follows: Firstly, from research stream of VBCs, this paper focuses on firms’ feedbacks. Previous research mainly focused on user interactions, neglecting firms’ critical role in “educating” and guiding the community users’ behaviors. This paper is a valuable supplement to the existing research regarding the influences of firms’ feedbacks frequency and text length on users’ posting behaviors. Secondly, previous research is dedicated to conventional variables such as e-WOM and intention to purchase, but not enough attention has been paid to matching linguistic styles in VBCs (Xiang et al., 2022). This paper also opens up new ideas for future research on VBCs, as LSM in VBCs is vital for cultivating a shared sense of community (Ludwig et al., 2014; Zhang et al., 2021) and even for further community prosperity. Third, relatively few empirical studies on secondary data on how to dynamic change LSM in VBCs. Based on the firm’s action perspective, this paper is the pioneer in empirically studying the LSM by secondary data, expanding the current methodological scope of LSM research. Fourth, from the perspective of firm practice, the marketing practice of VBCs is increasingly becoming a new marketing option for firms when facing the COVID-19 epidemic (Shin and Perdue, 2022). However, research with secondary data is relatively scarce compared to the rapidly rising practice of VBCs. This paper uses nearly 70,000 copies of MIUI community data as the unit of analysis, and the results of this study also provide valuable lessons for firms’ VBCs management practices.

## Literature and hypotheses

### Communication accommodation theory

Giles et al. (1973) found that people would adapt their accents to each other in conversation to make it more harmonious and smooth (Giles et al., 1973) and formally proposed the theory of interpersonal accommodation based on this everyday phenomenon. Subsequent research has expanded on this theory and has shown that this mutual accommodation between individuals occurs in speech, pronunciation, postures, and other behaviors. For example, the diction style of a student’s written responses subconsciously approximates the diction style of the question (Ireland and Pennebaker, 2010). Giles et al. (1987) extended the interpersonal accommodation theory to explain the adaptive behaviors of individuals in their communication process and proposed the communication accommodation theory, referring that people adjust (or accommodate) their language style to one another in the communication process to increase efficiency between both parties and maintain a positive identity. In the communication process, individuals use three linguistic strategies: convergence, divergence, and maintenance (Gnisci, 2005). Convergence refers to the communicator becoming more like the other communicator in terms of language (e.g., speed of speech, accent), paralanguage (e.g., pauses), and non-verbal features (e.g., smiles), while divergence refers to the opposite of convergence, where the communicator becomes less like the other communicator in these cases. Correspondingly, the maintenance strategy refers to the communicator keeping his or her communication behaviors intact. According to communication accommodation theory, all three strategies are adaptive changes in the direction of the communication partner, and individuals may choose different directional communication strategies for different motivations.

Recently, scholars have begun to use communication accommodation theory to explain virtual interaction in the online environment. For example, Presbitero (2021) applied this theory to depict the communication accommodation process of global virtual teams. Deng et al. (2021) found that brand post linguistic styles influence consumer engagement behaviors (i.e., like, share, and comment) as a communication accommodation process. While communication accommodation theory is often used to explain individuals’ verbal interactions in the real world, its application in virtual environments is only beginning (Ludwig et al., 2014; Wang et al., 2019). Similar to Deng et al. (2021)’s research context (Facebook pages), this paper argues that the textual interactions (e.g., posts and feedbacks) between firms and users in VBCs can also be explained by communication accommodation theory.

### Linguistic style matching

Linguistic style matching, which measures the degree of integration or synchronization between communication partners (Ireland and Pennebaker, 2010), is an algorithm developed mainly to calculate textual cohesiveness based on a computer-aided automated TA of words (Tausczik and Pennebaker, 2010; Wang et al., 2022) and a pivotal point for understanding communication conformity. It refers to the degree of similarity in the linguistic styles of the two parties in a communication process. Existing research suggests that a person adapts his or her linguistic style to others when interacting with different people (Niederhoffer and Pennebaker, 2002). According to Byrne’s (1971) similarity-liking hypothesis, LSM could result in a stronger link between the conversation participants, predicting their attitudes and behaviors. When both parties in a conversation are more actively engaged, the higher the LSM of both parties. At the same time, LSM can reveal the behaviors of both parties in a communication. For example, Ireland et al. (2011) found that individuals prefer to date someone with a linguistic style close to their own and that the more similar the linguistic style, the longer the relationship between the two parties. Ludwig et al. (2013) found that if the linguistic style of the product interest group matched the linguistic style of the product review, the higher the product’s conversion rate. The higher the overall level of LSM between the users and the community, the more actively the users will engage with the community; that is, they will post more and improve the content quality (Ludwig et al., 2013). LSM between an individual review and all reviews for the same product category could be a diagnostic cue for review helpfulness (Wang et al., 2019). One possible mechanism why LSM can reveal or predict people’s behavior is that when individuals have a higher LSM with others, they are closer to others socially and develop a higher level of identification with them (Ireland and Pennebaker, 2010; Topaloglu and Dass, 2021).

Given the importance of LSM in various business discourse texts, some studies began to probe what drives the formation of LSM. For example, the online reviews’ sentiment affects the degree of synchrony of expressed texts made by recipients (Yin et al., 2016). Wang et al. (2022) found that the prioritized arrangement of words of importance contributes to the product-centered language style, an indicator calculated based on the LSM algorithm. However, current studies do not answer what firms can do to influence the LSM in the VBCs setting. In particular, how firms’ behaviors influence the LSM between the UGC and firm-generated content (for simplicity, we called LSM in the hypotheses paragraphs) in VBCs is still unexplored. Therefore, this paper explores and clarifies the outcomes, processes, and conditions under which firms’ feedbacks affect the LSM, which is undoubtedly beneficial for firms to manage VBCs and develop effective interactions with users.

### The effect of firms’ feedbacks on linguistic style matching

Virtual brand communities are where firms have the freedom to decide to provide or not provide feedbacks to users on their posts. It has been established that the firm’s feedbacking behaviors to users’ posted content attract users to keep posting content. For example, a study by Piezunka and Dahlander (2019) found that as long as firms were able to provide feedbacks to users, even if the firm’s feedbacks consisted of rejecting the users’ suggestions, then those users who received feedbacks from the firm would put their next post more quickly than those who did not receive feedbacks from the firm. Conversely, firms that do not provide feedbacks may leave users with the impression that the firm does not care what users say, even though they have created or are involved in the VBC (Alexy et al., 2012). Of the feedbacks provided by the firm, the more frequent the feedback, the better it is to demonstrate to users the firm’s intention to build and maintain a relationship with them (Piezunka and Dahlander, 2019). This is because the more often a firm gives feedbacks, the better it is for the users to feel that the firm is acting as a companion and friend to the users (Nambisan and Baron, 2010). In addition, if a firm provides feedbacks, this action indicates that the firm is allocating its limited management efforts to the users. For example, the firm is willing to pay attention to the content posted by average users and demonstrates the firm’s willingness to actively participate in the communication with these users (Chan et al., 2015). According to communication accommodation theory, when a community user communicates with the firm, he or she expects the other party (the firm) to participate in the communication process actively (Giles, 2016). Therefore, the more feedbacks from firms, the more users will change their linguistic style to get more dialogs with firms. They will tend to make changes and adapt to the linguistic style of firms’ feedbacks when posting the following content. In summary, this study proposes the following hypothesis.

**Hypothesis 1:** The more firm’s feedbacks are given, the closer the linguistic style of the user’s following produced content will be to the linguistic style of the firm’s feedbacks. That is, the LSM will increase.

However, as the frequency of the firm’s feedbacks intensifies, the greater the chance that the users’ linguistic style will move closer to that of the firm’s feedbacks. This paper argues that the feedbacks text length may cause users’ linguistic styles to deviate from the firm’s feedbacks. This is because users in an online environment have a limited attention span and information processing capacity and prefer to keep their content concise (Frick et al., 2021). Especially on mobile devices, users prefer text messages that can be processed in a short time (Efremova et al., 2020). Therefore, except to comprehend the substantive information contained in the feedbacks, users need to be aware of and recognize the linguistic style of the feedbacks in the limited processing time. However, the firm’s feedbacks text length may increase users’ cognitive load, which may cause cognitive overload and reduce the users’ efficiency in learning the firm’s linguistic style in the feedbacks (Paas et al., 2003). Therefore, the longer the firms’ feedbacks text length, the more difficult it is for users to capture and imitate the current linguistic style of firms’ feedbacks. When posting the following content, the users’ linguistic style may deviate from the established linguistic style of firms’ feedbacks. Thus, the following hypothesis is proposed:

**Hypothesis 2:** The longer the firm’s feedbacks text length, the more the linguistic style of the users following posted content will deviate from the linguistic style of the firm’s feedbacks. That is, the LSM will decrease.

### The moderation of produced content’s characteristics

According to the communication accommodation theory, users have certain tendencies regarding the level of communication accommodation they should exhibit during the communication process (Giles, 2016). The emotional polarity of users’ posted content is often an indicator of their attitude toward a specific consumption experience and the willingness to continuously participate in the VBC (Jang and Moutinho, 2019), which results in a change in their communication conformity strategy. If a user’s existing posts are overall positive, the user is more willing to create a like-minded relationship with the VBC, i.e., understanding, adapting, and imitating the firm’s behaviors (Li et al., 2022). Accordingly, the influence of firms’ feedbacks frequency on the user’s imitation and convergence to the firm’s linguistic style may be accelerated. Similarly, when users are willing to maintain a relationship with a firm, they are also more willing to allocate more cognitive capacity and load to comprehend, integrate, and interpret the firm’s speech acts or behaviors, thus preparing for integration into the conversation with the firm (Bhattacharjee et al., 2022). Therefore, the sentiment of users’ produced content may play a moderating role in the influence process of firms’ feedbacks to LSM. The more positive the sentiment of the users’ existing textual content, the more it positively contributes to the positive effect of the firms’ feedbacks frequency on the LSM of users’ following content and counteracts the negative effect of the firms’ feedbacks text length on LSM of users’ following content. Thus, the two hypotheses are formulated:

**Hypothesis 3:** If the sentiment of the users’ produced content is more positive, then the positive effect of the firms’ feedbacks frequency on the LSM is stronger. That is, the sentimental valence of the users’ produced content positively moderates the relationship between the firms’ feedbacks frequency and the LSM of users’ following content.

**Hypothesis 4:** If the sentiment of the users’ produced content is more positive, then the negative effect of the firms’ feedbacks text length on the LSM is weaker. That is, the sentimental valence of the users’ produced content negatively moderates the relationship between the firms’ feedbacks text length and the LSM of users’ following content.

In VBCs, users may compare’ the efforts of both parties (e.g., the firm and themselves) in the communication process and decide their linguistic strategies based on comparison results (Santos, 2022). In other words, when posting new content, users balance their effort with those of the firm and strategically design the text of their posts to match the other party’s effort in the exchange process. The longer the total text length of the user’s posted content, the more effort the user has put into the interaction process with the firm (Santos, 2022). According to fairness heuristic theory, individuals will use themselves as a reference point to determine whether something is fair or not based on their own experiences (Jordan et al., 2022). Therefore, if a user puts more effort into exchanging with a firm, the users will also expect the firm to put in enough reciprocal effort. Users will use the total text length of their produced content as a reference point to judge the level of the firm’s effort embedded in the feedbacks content (Aras et al., 2022) and then adjust their behaviors based on this judgment. The feedbacks frequency and text length represent two types of effort, the former reflecting the times or width of effort and the latter indicating the depth of effort. The depth of effort reflects a deeper level than the times of effort and plays a more significant role in inferring the users’ perception of the firms’ effort.

This paper infers that the total text length of a user’s posted content, as a representation reflecting the user’s effort in the VBC, moderates the influence of the firms’ feedbacks on the LSM of users’ following content. Specifically, when the user’s effort is high, the user will be insensitive to the firm’s effort (Söderlund and Sagfossen, 2017). Therefore, when the firms’ feedbacks frequency intensifies, users will perceive fewer firms’ efforts and thus have less motivation to match firms’ linguistic styles. For example, if a user posts 1000 words or more every day, if the firm’s response rate is once a day, the user is obviously disgruntled as the mismatch of the two parties’ efforts. Meanwhile, users will also compare the firms’ feedbacks text length with their own posted content. When the users’ text length is longer, the users’ threshold for processing the firms’ feedbacks will also increase as they have already developed the ability to collect, produce, and read complex text (e.g., long feedbacks). Thus, the adverse effect of firms’ feedbacks text length on the users’ cognitive load will decrease or even diminish, thereby counteracting the negative effect of firms’ feedbacks text length on the LSM of users’ following content. Therefore, the following hypotheses are proposed.

**Hypothesis 5:** If the total text length of user-produced content is long, then the positive effect of the firms’ feedbacks frequency on LSM is weaker. That is, the text length of produced content can weaken the positive effect of the firms’ feedbacks frequency on the LSM of users’ following content.

**Hypothesis 6:** If the total text length of user-produced content is long, then the negative effect of the firms’ feedbacks text length on LSM is weaker. That is, the text length of produced content can offset the negative effect of firms’ feedbacks text length on the LSM of users’ following content.

## Materials and methods

### Data

The original data used in this paper comes from the discussion forum under MIUI, an online community established by Xiaomi in 2010 for users to discuss MIUI, Xiaomi’s mobile phone operating system. The discussion forum is one of the subsections. In this paper, we used a web crawler to crawl the data of users’ posts in this forum and specifically collected data in the following two aspects: First, post data, including the identity of posters (whether firm feedbacks), posting time, and post content. Secondly, the posts’ feedbacks data, including the identity of feedbackers, posting time, and feedback content. According to the identity of the feedbackers, this paper divided the feedbacks into other users’ and the firm’s feedbacks. Considering that the VBC was established in 2010 and experienced rapid growth in the early stage of the forum, the time range captured in this paper is from January 1, 2013 to December 31, 2014. However, Xiaomi has made two significant upgrades to its MIUI system during this period, MIUI V5 was released on April 9, 2013, and MIUI 6 was released on August 16, 2014. To avoid possible potential impacts before and after the new system’s release, this paper selected data from 2 months after the release of MIUI V5 to 2 months before the release of MIUI 6, a total of 373 days. The minimum unit of time in this paper is a month. So the date of a total of 13 months was recorded for each user’s posts in each month, and the firm’s feedbacks the user received each month. In the original data, the total number of observations was 69463, corresponding to a total number of 9777 users, but 7252 had no more than one post. Although many users in the MIUI forum are not active in the number of posts, the literature has noted that this phenomenon is common in online communities and does not affect the analysis results (Huang et al., 2014).

### Measurements

In order to calculate the degree of LSM, a reasonable calculation method must be constructed. LSM is an algorithm developed mainly to calculate verbal cohesiveness based on an automated TA of words, and this computer-aid text analysis can be more accurate in judging the relative distance between words than other methods (e.g., manual coding or experiment-based LSM). It is important to note that the LSM is only calculated for functional words. The reason for this is that non-functional words (e.g., nouns, adjectives) reflect the content of the text, while functional words (e.g., pronouns, quantifiers) reflect the style of the text (Tausczik and Pennebaker, 2010). The LSM itself refers to the matching of linguistic styles between the communicating parties and does not involve the content of the text, so it is more reasonable to use functional words.

Followed by the method proposed by Xiang et al. (2022), this paper draws on the ideas of Ireland and Pennebaker (2010), combining Jieba lexical analysis with the Dictionary of Modern Chinese Functional Words to classify functional words into seven categories: auxiliary words, adverbs, prepositions, conjunctions, pronouns, orientation words, and tone words. The specific text splitting and lexical recognition of the splitting results were done using Python’s third-party library jieba library. Referring to Ludwig et al. (2014), the LSM is calculated as follows, taking two texts, A and B, as an example: First, the proportion of each of the seven types of functional words in text A and text B is calculated, for example, the proportion of auxiliary words in each of text A and text B is


(1) $$\begin{array}{c} F\,W\,C_{A,\text{aux}}=\frac{\text{A}\,\mathrm{ux}\,\mathrm{words}\,\mathrm{in}\,A}{\text{Total}\,\text{words}\,\text{in}\,A},\text{and}\\ F\,W\,C_{B,\text{aux}}=\frac{\text{A}\,\mathrm{ux}\,\mathrm{words}\,\mathrm{in}\,\text{B}}{\text{Total}\,\text{words}\,\text{in}\,B} \end{array}$$

Then, the LSM of text A and text B on these seven types of functional words are calculated. e.g., the LSM of Text A and Text B on the auxiliary words is


(2) $$L\,S\,M_{A\,B,\text{aux}}=1-\frac{\left|F\,W\,C_{A,\text{aux}}-F\,W\,C_{B,\text{aux}}\right|}{F\,W\,C_{A,\text{aux}}+F\,W\,C_{B,\text{aux}}+0.0001}$$

Adding 0.0001 to the denominator is to prevent a denominator of 0. In addition, it can also be seen from Eq. 2 that the value of LSM ranges from 0 to 1. The higher the LSM, the better the match between the linguistic style of text A and text B.

Finally, the LSM between text A and text B is the mean value of the LSM over these seven classes of functional words. That is


(3) $$L\,S\,M_{A\,B}=\frac{1}{7}\,\left(L\,S\,M_{A\,B,\text{aux}}+L\,S\,M_{A\,B,\text{adv}}+\ldots +L\,S\,M_{A\,B,\text{modal}}\right)$$

Referring to the treatment by Ludwig et al. (2014), for monthly postings of two or more, the texts are treated as belonging to the same text, only from different paragraphs of that text. Similarly, the LSM between the firm’s feedbacks and users’ posts and between other users’ feedbacks and users’ posts were calculated based on distinguishing whether the feedbacks text came from the firm or other users.

### Models and variables

Given the panel form of the data, this paper uses a panel fixed effects model to validate H1 to H6 with the following model as this model can exclude the “noises” of unobserved factors (Wooldridge, 2008) and has been applied in many quantitative studies (e.g., Capaldi et al., 2014). This paper will use Eq. 4 to test all the hypotheses.


$${\text{f\_lsmf}}_{i,t+1}=\ln{\text{firm\_fknum}}_{i,t}+\ln{\text{firm\_fklen}}_{i,t}+{\text{ptemo}}_{i,t}$$

$$+\ln{\text{ptlen}}_{\text{t}}+{\text{ptemo}}_{i,t}\times \ln{\text{firm\_fknum}}_{i,t}$$

$$+{\text{ptemo}}_{i,t}\times \ln{\text{firm\_fklen}}_{i,t}+\ln{\text{ptlen}}_{i,t}$$

$$\times \ln{\text{firm\_fknum}}_{i,t}\,\ln{\text{ptlen}}_{\text{t}}\times \ln{\text{firm\_fklen}}_{i,t}$$

(4) $$+{\text{C}}_{i,t}+\mathrm{Fixed}\,\mathrm{Effect}\,{\text{u}}_{i,t}$$

In Eq. 4, i denotes individuals, t denotes months, future_lsmf_i,t + 1_ denotes the LSM between the user’s posts and firm’s feedbacks in t + 1 month, C_i,t_ denotes other control variables. The Fixedeffect contains individual fixed effect and time fixed effect. The u_i,t_ is the random disturbance term, and all variables are described in Table 1.

**TABLE 1:** Description of variables and descriptive statistics.

Variables	Meanings	Mean	SD	Min.	Max.
*future*_*lsmf*_*i*,*t* + 1_	The LSM score between all posts produced by user *i* in month *t+1* and the firm’s feedbacks before month *t+1*.	0.467	0.174	0.001	0.986
*lnfirm*_*fknum*_*i*,*t*_	To month *t*, the firm’s feedbacks frequency received by user *i* (ln form).	0.423	0.515	0.000	4.489
*lnfirm*_*fklen*_*i*,*t*_	To month *t*, the firm’s feedbacks text length received by user *i* (ln form).	4.113	1.031	0.693	8.592
*ptemo*_*i*,*t*_	To month *t*, the sentimental mean of all the posts of users *i*.	0.245	0.525	−1.000	1.000
*lnptlen*_*i*,*t*_	To month *t*, the text length of all the posts of users *i* (ln form).	4.66	1.141	0.693	10.298
*lnculpt*_*i*,*t*_	To month *t*, the number of posts produced by user *i* (ln form).	0.863	0.379	0.693	5.308
*lncomt*_*given*_*i*,*t*_	To month *t*, the number of the feedbacks given by user *i* to other posts (ln form).	0.477	0.892	0.000	7.182
*past*_*lsmf*_*i*,*t*_	To month *t*, the LSM score of user *i*′s posts and the firm’s feedbacks.	0.537	0.178	0.001	1.000
*firm*_*kemo*_*i*,*t*_	To month *t*, the sentimental mean of the firm’s feedbacks received by user *i*.	0.220	0.560	−1.000	1.000
*past*_*lsmu*_*i*,*t*_	To month *t*, the LSM score of user *i*′s posts and other users’ feedbacks.	0.526	0.170	0.001	1.000
*lnuser*_*fknum*_*i*,*t*_	To month *t*, the other users’ feedbacks frequency received by user *i* (ln form).	1.672	0.990	0.000	6.94
*lnuser*_*fklen*_*i*,*t*_	To month *t*, the other users’ feedbacks text length received by user *i* (ln form).	4.843	1.387	0.693	10.524
*user*_*emo*_*i*,*t*_	To month *t*, the sentimental mean of other users’ feedbacks received by user *i*.	0.230	0.408	−1.000	1.000

## Hypotheses testing

### Descriptive statistics

Firstly, the paper presents descriptive statistics for all variables (Table 1). From the table, it is easy to see the mean and standard deviation of the variables. Some of the variables are logarithmic, but the differences between their logarithmic minimum and maximum values are still relatively small. After taking the logarithmic form, the mean and standard deviation differences are not significant enough. Thus, it allows further analysis.

### Empirical results

This paper builds two models separately to verify the predicted power of interaction terms. Model 1 only contains main variables without interactions, and model 2 is the full model with all interactions. The regression results are shown in Table 2. According to model 1, the firm’s feedbacks frequency and text length did not significantly affect the LSM, indicating that the firm’s feedbacks model (model 1) alone did not have predictive power on users’ following posting behaviors, and additional variables need to include the model. However, the predicted power was shown when all the interaction terms were added to model 2. The coefficient of *ln*⁡firm_fklen is significantly negative, indicating the total firms’ feedbacks text length has a significant negative effect on the LSM, which means that the longer the total text length of firms’ feedbacks, the more users tend to express a different linguistic style than the firm’s feedbacks when releasing new posts, and therefore H2 is supported. In a similar vein, the coefficient of the firm’s feedbacks frequency (*ln*⁡firm_fknum) is significantly positive, supporting H1.

**TABLE 2:** Regression results.

Meanings	Variables	Model 1	Model 2
The firm’s feedbacks frequency received by focal user.	*lnfirm*_*fknum*	−0.020 (0.040)	0.222* (0.117)
The firm’s feedbacks text length received by focal user.	*lnfirm*_*fklen*	−0.001 (0.018)	−0.152** (0.060)
The sentimental mean of all the posts of focal user.	*ptemo*	0.068 (0.043)	−0.200 (0.125)
The text length of all the posts of focal user.	*lnptlen*	0.006 (0.019)	−0.050* (0.026)
The number of posts produced by focal user.	*lnculpt*	−0.002 (0.045)	−0.011 (0.046)
The number of the feedbacks given by focal user.	*lncomt*_*given*	−0.008 (0.013)	−0.003 (0.013)
The LSM score of focal user’s posts and the firm’s feedbacks.	*past*_*lsmf*	0.028 (0.055)	0.031 (0.056)
The sentimental mean of the firm’s feedbacks received by focal user.	*firm*_*fk*emo	0.039 (0.032)	0.036 (0.032)
The LSM score of focal user’s posts and other users’ feedbacks.	*past*_*lsmu*	0.046 (0.071)	0.036 (0.070)
The other users’ feedbacks frequency received by focal user.	*lnuser*_*fknum*	−0.049 (0.038)	−0.043 (0.038)
The other users’ feedbacks text length received by focal user.	*lnuser*_*fklen*	0.044** (0.021)	0.041* (0.021)
The sentimental mean of other users’ feedbacks received by focal user.	*us*_*fk*emo	−0.026 (0.046)	−0.028 (0.045)
The interaction of focal user’s sentiment and firm’s feedbacks frequency.	*ptemo*×*lnfirm*_*fknum*		−0.086 (0.068)
The interaction of focal user’s sentiment and firm’s feedbacks length.	*ptemo*×*lnfirm*_*fklen*		0.083** (0.038)
The interaction of focal user’s text length and firm’s feedbacks frequency.	*lnptlen*×*lnfirm*_*fknum*		−0.034** (0.016)
The interaction of focal user’s text length and firm’s feedbacks length.	*lnptlen*×*lnfirm*_*fklen*		0.021** (0.009)
–	_cons	0.341*** (0.110)	0.763*** (0.189)
–	Individual FE	Yes	Yes
–	Time FE	Yes	Yes
–	*N*	1,752	1,752
–	R^2^	0.050	0.063

*p < 0.1, **p < 0.05, ***p < 0.001.

SE in brackets, FE means fixed effect, ln prefix means that the variable takes the logarithm, the same as below.

In addition, according to Model 2, the interaction term between the sentiment of the user’s produced content and the firm’s feedbacks text length (ptemo×*ln*⁡firm_fklen) is significantly positive, supporting H4. The interaction term between the length of the user’s produced content and the firm’s feedbacks frequency (*ln*ptlen×*ln*⁡*firm*_*fknum*) is significantly negative, supporting H5. Finally, according to Model 2, the interaction term between the user’s produced content total length and the firm’s feedbacks text length (*ln*ptlen×*ln*⁡*firm*_*fklen*) is significantly positive, supporting H6. In summary, of the six hypotheses in this paper, only H3 is not supported, while the rest of the hypotheses are supported.

### Robustness checks

While most of the hypotheses in this paper are supported, further robustness tests are still needed. Firstly, there is the issue of endogeneity. As the dependent variable is required to be observed after users have posted, there may be a problem of user self-selection bias, i.e., whether users post or not is an auto-selective variable. Secondly, there is the issue of heterogeneity. The above regression results are for the entire sample, but for VBCs, most users are not active, while the minority of users are more active. Therefore whether the above results hold for the active minority user group vs. the inactive majority user group must be further checked.

### Self-selection bias

This paper may have an endogeneity problem due to sample self-selection. This is because we observe a sample of only those users who post, and do not observe users who do not post. In order to address the endogeneity problem caused by the self-selection bias of whether users post or not, a Heckman two-step method is used to detect this issue (Wooldridge, 2008). The Heckman two-step method provides a means of correcting for non-randomly selected samples and has been used in many quantitative studies (e.g., Lafuente et al., 2019) because of its simplicity and practicality. In the first step, a probit model is first built to estimate the inverse mills ratio (IMR), which controls the self-selection bias (Wooldridge, 2008). The IMR is added to Eq. 4 for regression in the second step. The model developed in the first step is shown below.


$${\text{y}}_{i,t+1}∼r\,e\,g\,i\,s\,t\,e\,r_{i}+l\,n\,f\,i\,r\,m\,\_\,f\,k\,n\,u\,m_{i,t}+l\,n\,f\,i\,r\,m\,\_\,f\,k\,l\,e\,n_{i,t}+p\,t\,e\,m\,o_{i,t}$$

(5) $$+l\,n\,p\,t\,l\,e\,n_{t}+C_{i,t}+T\,i\,m\,e\,F\,i\,x\,e\,d\,E\,f\,f\,e\,c\,t+\alpha_{i,t}$$

In the Eq. 5,_yi,t + 1_ is a 0/1 dummy variable, and 1 means the user i puts a post in t + 1. Similar, register_i_ indicates the registered time of individual user i, the earlier the registration time, the smaller this number is. Also, α_i,t_ denotes the random perturbation term. The Heckman two-step method is used in this paper (Heckman, 1979). The first step is to estimate the posting probability according to all independent variables and then calculate IMR through a probit model. In the second step, IMR is substituted into the regression equation. The specific regression results are shown in Table 3.

**TABLE 3:** Regression results after controlling for self-selection bias.

Meanings	Variables	Model 3 (Stage 1)	Model 4 (Stage 2)
Register or not	*register*	0.003 (0.002)	
Inverse mills ratio	*IMR*		0.435** (0.216)
The firm’s feedbacks frequency received by focal user.	*lnfirm*_*fknum*	0.015 (0.075)	0.292** (0.118)
The firm’s feedbacks text length received by focal user.	*lnfirm*_*fklen*	−0.057** (0.026)	−0.183*** (0.060)
The sentimental mean of all the posts of focal user.	*ptemo*	0.057 (0.043)	−0.193 (0.126)
The text length of all the posts of focal user.	*lnptlen*	0.003 (0.022)	−0.042 (0.027)
The number of posts produced by focal user.	*lnculpt*	0.714*** (0.071)	0.232* (0.137)
The number of the feedbacks given by focal user.	*lncomt*_*given*	0.223*** (0.023)	0.072* (0.039)
The LSM score of focal user’s posts and the firm’s feedbacks.	*past*_*lsmf*	−0.261** (0.114)	−0.056 (0.068)
The sentimental mean of the firm’s feedbacks received by focal user.	*firm*_*fk*emo	0.003 (0.037)	0.036 (0.031)
The LSM score of focal user’s posts and other users’ feedbacks.	*past*_*lsmu*	−0.025 (0.124)	0.023 (0.070)
The other users’ feedbacks frequency received by focal user.	*lnuser*_*fknum*	−0.185*** (0.048)	−0.108** (0.052)
The other users’ feedbacks text length received by focal user.	*lnuser*_*fklen*	0.112*** (0.030)	0.080*** (0.030)
The sentimental mean of other users’ feedbacks received by focal user.	*user*_*fk*emo	0.067 (0.056)	−0.003 (0.046)
The interaction of focal user’s sentiment and firm’s feedbacks frequency.	*ptemo*×*lnfirm*_*fknum*		−0.094 (0.068)
The interaction of focal user’s sentiment and firm’s feedbacks length.	*ptemo*×*lnfirm*_*fklen*		0.088** (0.039)
The interaction of focal user’s text length and firm’s feedbacks frequency.	*lnptlen*×*lnfirm*_*fknum*		−0.044*** (0.016)
The interaction of focal user’s text length and firm’s feedbacks length.	*lnptlen*×*lnfirm*_*fklen*		0.023** (0.009)
–	_cons	−1.686*** (0.195)	−0.175 (0.538)
–	Individual FE	NA	Yes
–	Time FE	Yes	Yes
–	*N*	25,790	1,752
–	R^2^	NA	0.069

*p < 0.1, **p < 0.05, ***p < 0.01.

According to Model 4 (Stage 2) in Table 3, the coefficient on IMR is significant; therefore, the sample has a severe self-selection problem. When the IMR is used to control for user self-selection bias, the coefficient of *ln*⁡firm_fknum is still significant, indicating that the higher the total number of firm’s feedbacks, the closer the linguistic style of new posts posted by users is to the linguistic style of the firm’s feedbacks, and therefore H1 is supported. Similarly, controlling for self-selection bias, the regression coefficients of four interaction terms did not change, corresponding to H2, H4, H5, and H6. Thus, several of the main hypotheses are supported, and the results are robust.

### Heterogeneity analysis

Since UGC is the core focus, this paper defines active or inactive users in terms of the amount of UGC. In this paper, we conducted a descriptive statistical analysis of the cumulative number of posts (culpt ) made by MIUI users, and the total number of posts for 93.60% of the observations was less than 4. Accordingly, a threshold of 4 was used to define the active and inactive users. If culpt is less than 4, the users are inactive, and if culpt is greater than 4, the users are active. The results of the heterogeneity analysis are shown in Table 4.

**TABLE 4:** Results of heterogeneity analysis.

Meanings	Variables	Model 5	Model 6
		*culpt* < 4	*culpt*≥4
Inverse mills ratio	IMR	−0.468 (4.628)	0.387* (0.230)
The firm’s feedbacks frequency received by focal user.	*lnfirm*_*fknum*	0.561 (0.822)	0.250** (0.127)
The firm’s feedbacks text length received by focal user.	*lnfirm*_*fklen*	−0.258 (0.318)	−0.181*** (0.065)
The sentimental mean of all the posts of focal user.	*ptemo*	−0.385 (0.422)	−0.407*** (0.139)
The text length of all the posts of focal user.	*ptlen*	−0.067 (0.129)	−0.078** (0.030)
The number of posts produced by focal user.	*lnculpt*	−0.145 (2.851)	0.139 (0.140)
The number of the feedbacks given by focal user.	*lncomt*_*given*	−0.067 (0.922)	0.059 (0.040)
The LSM score of focal user’s posts and the firm’s feedbacks.	*past*_*lsmf*	0.037 (1.036)	−0.016 (0.079)
The sentimental mean of the firm’s feedbacks received by focal user.	*firm*_*fk*emo	−0.270** (0.137)	0.058 (0.038)
The LSM score of focal user’s posts and other users’ feedbacks.	*past*_*lsmu*	0.169 (0.181)	0.012 (0.074)
The other users’ feedbacks frequency received by focal user.	*lnuser*_*fknum*	−0.083 (0.732)	−0.028 (0.052)
The other users’ feedbacks text length received by focal user.	*lnuser*_*fklen*	−0.021 (0.436)	0.073** (0.034)
The sentimental mean of other users’ feedbacks received by focal user.	*user*_*fk*emo	−0.051 (0.278)	−0.019 (0.062)
The interaction of focal user’s sentiment and firm’s feedbacks frequency.	*ptemo*×*lnfirm*_*fknum*	−0.208 (0.385)	−0.124 (0.081)
The interaction of focal user’s sentiment and firm’s feedbacks length.	*ptemo*×*lnfirm*_*fklen*	0.121 (0.100)	0.137*** (0.042)
The interaction of focal user’s text length and firm’s feedbacks frequency.	*lnptlen*×*lnfirm*_*fknum*	−0.056 (0.144)	−0.038** (0.018)
The interaction of focal user’s text length and firm’s feedbacks length.	*lnptlen*×*lnfirm*_*fklen*	0.020 (0.039)	0.022** (0.010)
–	_cons	0.763*** (0.189)	−0.175 (0.538)
–	Individual FE	Yes	Yes
–	Time FE	Yes	Yes
–	*N*	573	1179
–	R^2^	0.063	0.069

*p < 0.1, **p < 0.05, ***p < 0.01.

The results of this study are influenced by user heterogeneity. When the cumulative number of posts (culpt ) is less than 4, H1 to H6 are not supported. However, when the cumulative number of posts (culpt ) is greater than or equal to 4, H1, H2, H4, H5, and H6 were all supported, in line with the findings above. This result suggests that the effect of firms’ feedbacks on LSM of UGC may be limited to the more active user groups and not significant for the inactive user groups. This result reflects that firms that attempt to take advantage of feedbacks in influencing the linguistic style of users’ posts should choose the groups carefully. Otherwise, it may not have the desired effect.

## Discussion

Based on communication accommodation theory, this paper reveals why users engage in language style matching from the firm’s perspective. Using 69,463 posts on the MIUI forum as the empirical object to measure the level of LSM of each user’s post to the firm’s linguistic style, this paper examines the effect of the firm’s feedbacks frequency and text length on it and form the following results: Firstly, the more feedbacks firms give to users’ posts, the more users will adopt a similar linguistic style as the firm in their subsequent posting behaviors (increase the LSM). Consequently, firms should respond to users’ posts frequently, which helps to create a “unified” language style in the VBC. However, the firm’s feedbacks total length negative influence the LSM, implying that the firm should respond to users’ posts with concise text; otherwise, it will reduce the LSM between two communication participators. Secondly, the users’ relevant characteristics moderate the influence of the firm’s feedbacks on the LSM. This paper found that the positive effect of the firm’s feedbacks on LSM is further enhanced when the user has posted more positive content or more words and sentences. This study makes a certain amount of contributions to the VBCs literature and LSM research. At the same time, some research findings can help firms manage their brand communities effectively and develop dialogs with community users.

### Theoretical contributions

The main contributions of this paper are: Firstly, this study effectively extends the scope of communication accommodation theory. While most previous studies have placed this theory in the context of real world interactions (e.g., Giles, 2016) or user-to-user communication (e.g., Jing et al., 2020), this paper extends it to user-firm verbal interactions in VBCs setting and explores the dynamic communication process. To best of our knowledge, this work is the first to examine the dynamic LSM using secondary behavioral panel data, which expands the applied scope of communication accommodation theory and effectively echoes the previous LSM research in studying the LSM from a actionable perspective (Niederhoffer and Pennebaker, 2002; Liu et al., 2019).

Secondly, much of the previous literature on VBCs has focused on the outcomes of UGC, neglecting how to motivate community users effectively. Only a few scholars have begun exploring the attendance of UGC in such communities (e.g., Yao et al., 2021). Furthermore, given the importance of common language style in the online environment (Wang et al., 2022), this paper focuses on how to drive convergence between users and firms in terms of linguistic style, enriching the theoretical research on VBCs.

Third, scholars have focused on variables such as relationship norms (e.g., Aggarwal, 2004) in the specific community governance literature but still fail to answer the content coupling mechanisms between users and firms in online communities. This paper is undoubtedly a helpful addition to the community governance and management literature by approaching the issue from the perspective of LSM.

Beside, this paper is based on a computer-aided automated TA of words. Recently, the computer-aided text analysis (CATA) and natural language processing (NLP) has been applied in analyzing the users’ content in virtual environment (e.g., Saura et al., 2022). The innovative use of the LSM algorithm in this study makes it possible to measure the similarity in linguistic styles between firms and users, which is a methodological contribution.

### Practical implications

The study also suggests the following strategies for firms to manage their VBCs and interact effectively with their users. Firstly, firms should pay attention to users’ posting behaviors in the VBC in real-time and try to respond to their posts in a timely manner, which would help form a common language. Also, concise and short sentences were required in replying to users’ posts as this way can help users capture the firm’s content in a low effort condition, leading the to “imitate” the firm’s linguistic style easily and effectively.

Secondly, the paper also found that some characteristics of users’ past postings moderate the relationship between firm’s feedbacks and LSM, requiring firms to ask users to actively participate in the community by encouraging them to post and use positive words or phrases when managing the community in the first place as firm’s actions could exert greater impact in reaching a harmonious language matching in the community. Firms could also deploy computer-aided text technology to detect users’ emotional state in their published posts and carry out different targeted strategies to different users. For example, for those who published positive content users, firms could identify different users and favor active users to become the “seed” community users, leading to a typical linguistic style across the VBC.

### Limitations and future directions

The limitations of this paper are: Firstly, this paper only uses the MIUI forum as the research object to investigate the influence of firms’ feedbacks on matching users’ linguistic styles. Similar phenomena also exist in other VBCs, such as the “Apple Style” prevalence in the Apple community. Further research can be conducted to verify the generality of the findings. Secondly, this paper uses machine learning to refine the LSM objects of users’ posts and verify the influence relationships of firms’ feedbacks on them, but the corresponding influence paths have not yet been studied in detail and need to be further explored future research. Third, although the panel fixed-effects model was used to avoid the potential unobserved shocks, some posts’ details were unknown to researchers. For example, A negative post may represent the poster’s dissatisfaction with a single interaction rather than the brand. Further studies could conduct more detailed analysis when refined data were available. Fourth, given that research on the impact of LSM antecedents has only just begun (e.g., Wang et al., 2022), we examined them from the perspective of firms’ feedbacks. Future scholars may also consider the impact of other firm behaviors on LSM. For example, whether the firm replies at different times affect users’ learning ability and thus LSM.

## Data availability statement

The original contributions presented in this study are included in the article/supplementary material, further inquiries can be directed to the corresponding author.

## Author contributions

All authors listed have made a substantial, direct, and intellectual contribution to the work, and approved it for publication.

## Conflict of interest

The authors declare that the research was conducted in the absence of any commercial or financial relationships that could be construed as a potential conflict of interest.

## Publisher’s note

All claims expressed in this article are solely those of the authors and do not necessarily represent those of their affiliated organizations, or those of the publisher, the editors and the reviewers. Any product that may be evaluated in this article, or claim that may be made by its manufacturer, is not guaranteed or endorsed by the publisher.
